# Impact of Radiofrequency Ablation-Induced Glisson’s Capsule-Associated Complications in Patients with Hepatocellular Carcinoma

**DOI:** 10.1371/journal.pone.0170153

**Published:** 2017-01-18

**Authors:** Toru Wakamatsu, Sadahisa Ogasawara, Tetsuhiro Chiba, Masayuki Yokoyama, Masanori Inoue, Naoya Kanogawa, Tomoko Saito, Eiichiro Suzuki, Yoshihiko Ooka, Akinobu Tawada, Osamu Yokosuka

**Affiliations:** Department of Gastroenterology and Nephrology, Graduate School of Medicine, Chiba University, Chiba, Japan; Texas A&M University, UNITED STATES

## Abstract

**Background:**

Radiofrequency ablation (RFA) is commonly used to locally treat hepatocellular carcinoma (HCC). However, when tumors are close to the Glisson’s capsule, RFA may induce injury in this region, complicating therapeutic efforts. We investigated the impact of RFA-induced Glisson’s capsule-associated complications on liver function and prognosis of HCC patients.

**Methods:**

We retrospectively reviewed our patient database and found 170 early-stage HCC patients treated via RFA from April 2004 to December 2012. We defined RFA-induced Glisson’s capsule-associated complication as lasting hepatic arterioportal (AP) fistula, major intrahepatic bile-duct dilatation (affecting two or more subsegments), or hepatic infarction. We also defined liver failure as initial occurrence of either total bilirubin increase (>3.0 mg/dL), uncontrolled ascites, or encephalopathy.

**Results:**

In our cohort, 15 patients had RFA-induced Glisson’s capsule-associated complications (incidence of related complications, with some overlap: lasting AP fistula, n = 9; major intrahepatic bile-duct dilatation, n = 7; and hepatic infarction, n = 2). The cumulative incidence of liver failure before stage progression was significantly higher and the median overall survival (OS) was significantly lower (52.3 months) in HCC patients with Glisson’s capsule-associated complications than in those without Glisson’s capsule-associated complications (95.0 months). In addition, multivariate analysis demonstrated that Glisson’s capsule-associated complication was a significant independent factor associated with OS.

**Conclusions:**

In this study, we have shown that early-stage HCC patients with RFA-induced Glisson’s capsule-associated complications may have higher risks in poor prognosis.

## Introduction

Approximately 700,000 people annually die due to hepatocellular carcinoma (HCC), and HCC is the third most common cause of cancer mortality [1]. Radiofrequency ablation (RFA) is frequently used for the local treatment of HCC [2–4]. It is the best treatment alternative in patients with early-stage HCC who are not eligible for surgical resection. RFA extends survival by >60 months [5].

A cohort study including 1,170 HCC patients reported an RFA complication rate of 2.2% and a mortality rate of 0.003% [5]. In addition, a recent review of 34 studies showed that major complications and mortality rate was 4.1% and 0.15%, respectively [6]. However, the contribution of RFA-related complications to HCC patient survival is unclear.

The Glisson’s capsule extends into the liver as sheaths around the hepatic bile-ducts, hepatic arteries, and portal veins. HCC lesions adjacent to the Glisson’s capsule may be affected by RFA, thus increasing the risk of complications such as intrahepatic bile-duct dilatation, hepatic arterioportal (AP) fistula, and hepatic infarction. Most of these complications are irreversible and may negatively affect liver function and prognosis. In other cancers, postoperative complications significantly diminish patient survival [7–9]. To our knowledge, only few reports have analyzed the long-term outcomes of RFA-related complications [10]. The aim of this study was to retrospectively evaluate the prognostic impact of RFA-induced Glisson’s capsule-associated complications in patients with early-stage HCC.

## Patients and Methods

### Patients

This study was approved by the Research Ethics Committees of Graduate School of Medicine, Chiba University (approval number 2,246). Informed consents of examinations and treatments were obtained from all of patients included in this study according to the policy of our institution. Patient records/information were anonymized and de-identified prior to analysis.

Medical records were retrieved for HCC patients treated at our institution. Patients enrolled in this study were selected using the following inclusion criteria at their initial RFA: (1) the presence of histologically confirmed or clinically diagnosed HCC; (2) presence of early-stage HCC (single hypervascular ≤50 mm HCC lesion or ≤3 hypervascular ≤30 mm HCC lesions without macrovascular invasion or extrahepatic metastasis); and (3) Child—Pugh A or B. We excluded patients using the following criteria: (1) no hypervascular HCC, (2) no contrast-enhanced computed tomography (CT) or magnetic resonance imaging (MRI) after >3 months of initial RFA treatment, or (3) liver-unrelated death within a year.

### RFA

Treatment strategies in our institution are based on the Japanese guidelines [11]. First, we investigated whether definitive treatment can be accomplished by surgical resection or whether RFA is an alternative to surgical resection. RFA was performed as described previously [12]. Briefly, the procedures were performed under real-time ultrasound guidance (Power Vision 8000, Aplio XV, Aplio XG, or Aplio 500; Toshiba, Tokyo, Japan) and a 17-gauge cooled-tip electrode (Cool-Tip; RF Ablation System, Covidien, Boulder, Colombia, CO). Under conscious sedation, an electrode was inserted and radiofrequency was delivered for 6–15 min for each lesion. As appropriate, intrapleural or intraperitoneal fluid infusion was performed before electrode insertion. We evaluated effectiveness via dynamic CT or MRI on the day after RFA. The treatment assessments were performed as published previously [12]. To judge if ablation was complete or not, we compared images taken before and after ablation. The definition of completely ablated was as follows: post-ablation CT or MRI indicated a non-enhanced area covering the lesion where the tumor was located prior to ablation, outlined with a safety margin in the surrounding liver parenchyma.

### Follow-up, assessment of complications, and definition of RFA-induced Glisson’s capsule-associated complication

During follow-up, tumor markers, including α-fetoprotein (AFP) and des-γ-carboxy prothrombin, were measured every 1–2 months, and dynamic CT or MRI was performed every 4–6 months. To evaluate complications, all patient CT or MRI images and medical records obtained from just before initial RFA to additional HCC treatment or last follow-up were reviewed by 2 hepatologists (T. W. and S.O. with 7 and 12 years of experience, respectively). We defined RFA-induced Glisson’s capsule-associated complication as (1) lasting AP fistula that persisted for >3 months, (2) major intrahepatic bile-duct dilatation (affecting two or more subsegments), or (3) hepatic infarction.

### Statistical analysis

Demographic and clinical characteristics, the rate of RFA-induced Glisson’s capsule-associated complications in each subsegment, and the proportion of cause of death were compared by the chi-square test or Fisher’s exact test, as appropriate. Univariate and multivariate logistic regression analyses were used to estimate the odds ratios for risk factors of RFA-induced Glisson’s capsule-associated complications per nodules. Perivascular HCC was defined as a tumor located <5 mm from the first-order or second-order branches of portal veins, intrahepatic arteries, or bile-ducts based on the previous study [13]. Kaplan-Meier plots were used to estimate cumulative liver failure before stage progression and overall survival (OS). Cumulative liver failure before stage progression was measured from the date of initial RFA until the onset date of liver failure, which was defined as initial occurrence of either total bilirubin increase (>3.0 mg/dL), uncontrolled ascites, or encephalopathy. The censoring date was defined as the last date of radiological follow-up before stage progression. OS was measured from the date of initial ablation until the date of death. The censoring date was defined as the date of last follow-up. Univariate and multivariate Cox proportional-hazard models were used to estimate the hazard ratios for risk factor predicting cumulative liver failure before stage progression and OS. *P* values of <0.05 was considered statistically significant. All statistical analyses were performed using the SPSS statistical software (v. 23; SPSS-IBM, Chicago, IL, USA).

## Results

### Patients’ characteristics

**Fig 1:**
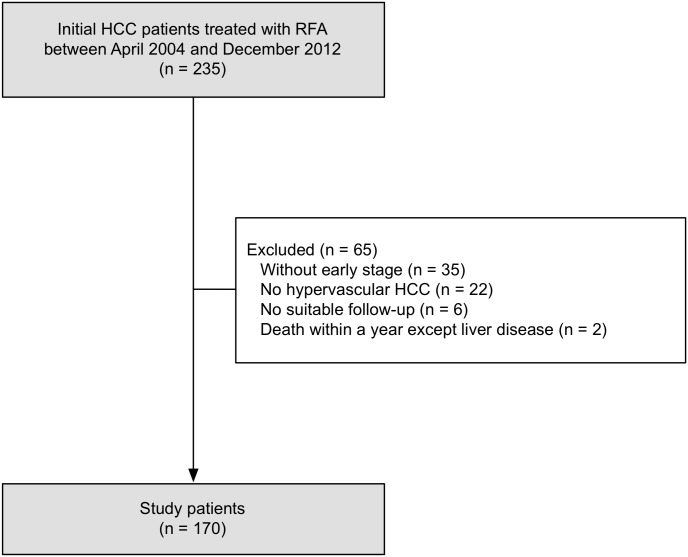
Study flow.

Of 235 patients who received RFA for initial HCC treatment between April 2004 and December 2012, 170 were eligible for inclusion in the present study (Fig 1). Sixty-five patients were excluded for the following reasons: 35 were not at the early stage, 22 lacked typical hypervascular HCC, 6 had no radiological assessment after ≥3 months of initial RFA, and 2 died of liver-unrelated disease within a year. The baseline characteristics of our eligible patients are summarized in Table 1. Median age was 74 years, and the most frequent etiology was hepatitis C virus (HCV, 76.5%), followed by hepatitis B virus (HBV, 7.1%). The median maximum tumor size was 19 mm (range, 6.5–42 mm), and 129 patients (75.9%) had a single tumor. One hundred and fifty-two patients were Child—Pugh A (89.4%) and the remaining were Child—Pugh B. Tumor size and liver function were similar to data in recent studies from Japan [14,15]. Procedural rate for HCC treatment was 1.35 (one procedure: 110 patients; two procedures: 60 patients). There was no significant difference between patients with and without RFA-induced Glisson’s capsule-associated complications.

**Table 1:** Baseline characteristics.

Variables	All cases	With RFA-induced Glisson’s capsule-associated complications	Without RFA-induced Glisson’s capsule-associated complications	*P*
**Gender** [*n* (%)]				
Male	114 (67.1)	11 (73.3)	103 (66.5)	0.776
Female	56 (32.9)	4 (26.7)	52 (33.5)	
**Age** [*n* (%)]				
≤74 years	92 (54.1)	10 (66.7)	82 (52.9)	0.307
>74 years	78 (45.9)	5 (33.3)	73 (47.1)	
**Child—Pugh class** [*n* (%)]				
A	152 (89.4)	14 (93.3)	138 (89.0)	0.510
B	18 (10.6)	1 (6.7)	17 (11.0)	
**HBs-Ag positive** [*n* (%)]				
Absent	158 (92.9)	14 (93.3)	144 (92.9)	0.714
Present	12 (7.1)	1 (6.7)	11(7.1)	
**HCV-Ab positive** [*n* (%)]				
Absent	40 (23.5)	4 (26.7)	36 (23.2)	0.488
Present	130 (76.5)	11 (73.3)	119 (76.8)	
**Alcohol abuse** [*n* (%)]				
Absent	156 (91.8)	13 (86.7)	143 (92.3)	0.356
Present	14 (8.2)	2 (13.3)	12 (7.7)	
**Maximum tumor size** [*n* (%)]				
≤20 mm	96 (56.5)	9 (60.0)	87 (56.1)	0.773
>20 mm	74 (43.5)	6 (40.0)	68 (43.9)	
**Number of liver tumors** [*n* (%)]				
Single	129 (75.9)	11 (73.3)	118 (76.1)	0.511
Multiple	41 (24.1)	4 (26.7)	37 (23.9)	
**AFP** [*n* (%)]				
≤100 ng/mL	142 (83.5)	12 (80.0)	130 (83.9)	0.463
>100 ng/mL	28 (16.5)	3 (20.0)	25 (16.1)	

Abbreviations: HBs-Ag, hepatitis B surface antigen; HCV-Ab, hepatitis C virus antibody; AFP, α-fetoprotein.

### RFA-induced Glisson’s capsule-associated complications in study patients

**Fig 2:**
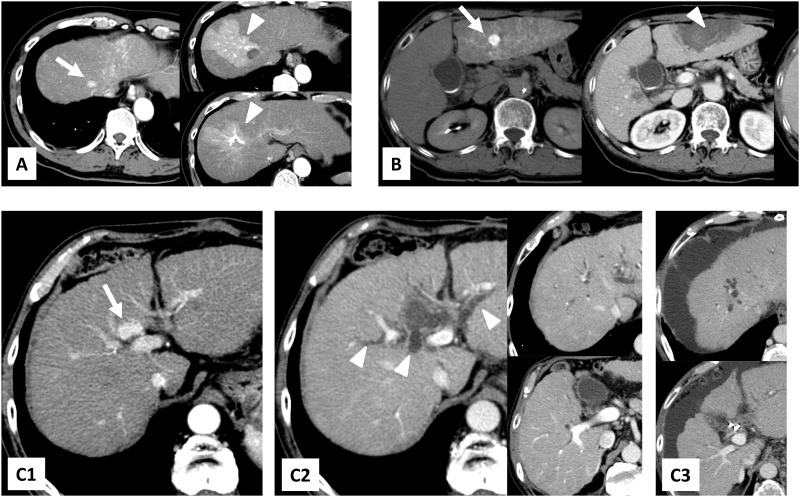
Typical cases of AP fistula (A), hepatic infarction (B), and bile-duct dilatation (C) after RFA in HCC patients. (A) A 66-year-old male with hepatic AP fistula after RFA for HCC in subsegment 8. Right: 8 months after RFA (Patient 12 in Table 3); (B) a 63-year-old female with liver infarction after RFA for HCC in subsegment 3. Middle: the next day of RFA. Right: 15 months after RFA (Patient 10 in Table 3); (C) a 68-year-old male with bile-duct dilatation in 2 subsegments and liver abscess after RFA for HCC in subsegment 4. C1: before RFA. C2: 4 months after RFA. C3: 2 years after RFA (Patient 11 in Table 3). White arrows show treated tumors and white arrowheads show RFA-induced Glisson’s capsule-associated complications.

In this study, AP fistula, intrahepatic bile-duct dilatation, and hepatic infarction due to initial RFA developed in 17 patients (10.0%), 14 patients (8.2%), and 2 patients (1.2%), respectively (Table 2). Fig 2 shows typical cases of AP fistula (A), hepatic infarction (B), and bile-duct dilatation (C) after RFA in HCC patients. Of the 17 patients who had AP fistula, 8 patients had transient AP fistulae and 9 patients had lasting AP fistulae, which were radiologically observed for 3 months or more after initial RFA. There were 7 patients with minor intrahepatic bile-duct dilatation (limited to one subsegment) and 7 patients with major intrahepatic bile-duct dilatation (affected two or more subsegments).

**Table 2:** Complications related to RFA in HCC patients.

Complications	N (%)
**AP fistula**	17 (10.0)
Transient	8 (4.7)
Lasting	9 (5.3)
**Intrahepatic bile-duct dilatation**	14 (8.2)
Minor	7 (4.1)
Major	7 (4.1)
**Portal vein thrombosis**	5 (2.9)
**Intra-abdominal hemorrhage**	4 (2.4)
**Hepatic infarction**	2 (1.2)
**Pleural hemorrhage**	2 (1.2)
**Hemobilia**	1 (0.6)
**Hepatic abscess**	1 (0.6)
**Nearby organ injury**	1 (0.6)
**Dissemination**	1 (0.6)
**AV fistula (transient)**	1 (0.6)

Abbreviations: AP, arterioportal; AV, arteriovenous

The details of 15 patients with RFA-induced Glisson’s capsule-associated complications according to the definition of this study are shown in Table 3. Three patients observed both intrahepatic bile-duct dilatation and AP fistula. Two of nine patients with lasting AP fistulae had fistulae distant from the ablation zone. Of the 7 patients with major intrahepatic bile-duct dilatations, 3 and 2 showed liver failure before stage progression and partial shrinkages of liver volume, respectively. Of the 9 patients with lasting AP fistulae, 3 had liver failure before stage progression. Partial shrinkages of liver volume were observed in 2 patients with liver infarctions.

**Table 3:** Patient list of RFA-induced Glisson’s capsule-associated complication in HCC patients.

Patients	Age	Gender	Maximum tumor size (mm)	Tumor number	Tumor location (subsegment)	Type of RFA-induced Glisson’s capsule-associated complication			Positional relation between ablation zone of tumor and Glisson's capsule-associated complication	Partial shrinkage of liver	Cause of liver failure before stage progression	Cause of death
						Bile-duct dilatation	AP fistula	Liver infarction				
1	74	F	15	1	S3	−	−	+	Nearby	+	−	Other disease
2	83	M	22	1	S3	+	−	−	Nearby	−	−	HCC
3	66	M	21	1	S8	−	+	−	Distant	−	Hepatic encephalopathy	Liver dysfunction
4	82	M	30	2	S8, S6	+	−	−	Nearby	−	−	Liver dysfunction
5	67	M	20	1	S7	+	+	−	Nearby	−	Jaundice	HCC
6	70	M	19	1	S8	−	+	−	Nearby	−	−	HCC
7	76	M	18	1	S4	−	+	−	Nearby	−	−	HCC
8	76	M	20	1	S4	+	+	−	Nearby	+	−	Alive
9	63	M	23	2	S6, S7	−	+	−	Nearby	−	Ascites	Alive
10	63	F	12	2	S3, S5	−	−	+	Nearby	+	−	Alive
11	68	M	21	1	S4	+	−	−	Nearby	+	Ascites	Liver dysfunction
12	66	M	13	1	S8	−	+	−	Nearby	−	−	Alive
13	72	F	18	2	S6, S8	−	+	−	Distant	−	−	Alive
14	72	M	24	1	S4	+	−	−	Nearby	−	Hepatic encephalopathy	Alive
15	75	F	12	1	S8	+	+	−	Nearby	−	−	Liver dysfunction

Abbreviations: F, female; M, male; S, segment; HCC, hepatocellular carcinoma; AP, arterioportal

### Impact of RFA-induced Glisson’s capsule-associated complications in HCC patients

S1 Table shows the rate of RFA-induced Glisson’s capsule-associated complication in each subsegment. Although there was no significant difference between subsegments, the incidences of Glisson’s capsule-associated complications were frequently observed in subsegment 3 and 4 (subsegment 3: 15.0%, subsegment 4: 15.4%). We analyzed the impact of risk factors of RFA-induced Glisson’s capsule-associated complications. In multivariate analysis, location of tumor defined as perivascular in this study (tumors located <5 mm from first-order or second-order branches of portal veins, intrahepatic arteries, or bile-ducts) was significant risk factor for RFA-induced Glisson’s capsule-associated complications (S2 Table).

**Fig 3:**
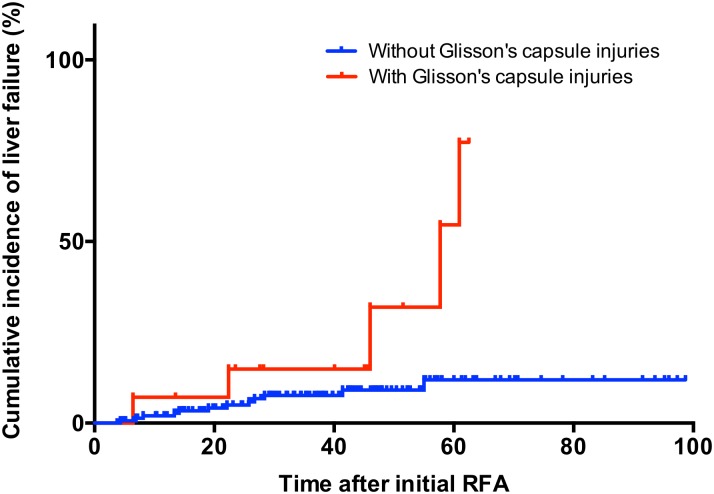
Cumulative incidence of liver failure before stage progression in RFA-treated patients.

Fig 3 shows Kaplan—Meier plots of cumulative liver failure before stage progression. Patients with RFA-induced Glisson’s capsule-associated complications had significantly higher incidence of liver failure before stage progression than those without Glisson’s capsule-associated complications (*P* = 0.003). Multivariate analysis indicated that Glisson’s capsule-associated complication was an independent risk factor for liver failure before stage progression as well as Child—Pugh B (Table 4).

**Table 4:** Univariate and multivariate analysis of cumulative liver failure before stage progression in RFA-treated HCC patients.

Variables	Univariate analysis		*P*	Multivariate analysis		*P*
	Hazard ratio	95% CI		Hazard ratio	95% CI	
**Gender, male**	2.384	0.684–8.303	0.172			
**Age, >74 years**	0.439	0.142–1.355	0.152			
**HBs-Ag positive**	0.451	0.058–3.472	0.444			
**HCV-Ab positive**	0.867	0.304–2.466	0.788			
**Alcohol abuse**	1.336	0.305–5.851	0.701			
**Child—Pugh B**	14.364	5.508–37.461	<0.001	27.263	8.567–86.761	<0.001
**Tumor size and number, >20 mm, multiple**	1.721	0.606–4.890	0.308			
**AFP, > 100 ng/mL**	0.662	0.151–2.902	0.585			
**RFA-induced Glisson’s capsule-associated complication**	4.360	1.534–12.389	<0.001	11.711	3.310–41.438	<0.001
**Complications except RFA-induced Glisson’s capsule-associated complication**	1.567	0.449–5.470	0.481			

Abbreviations: HBs-Ag, hepatitis B surface antigen; HCV-Ab, hepatitis C virus antibody; AFP, α-fetoprotein

**Fig 4:**
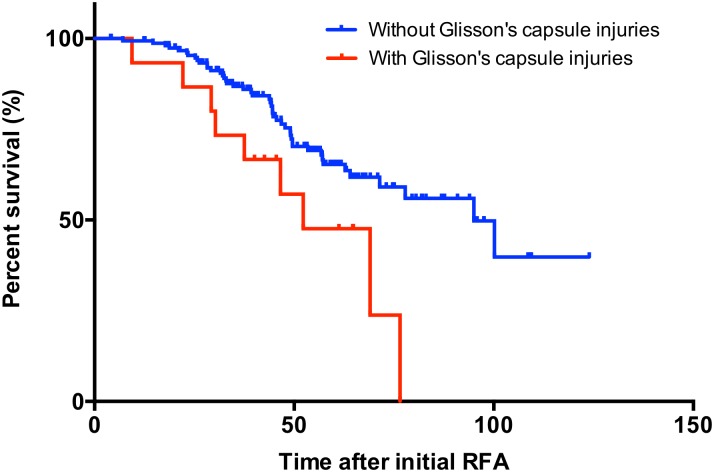
Overall survival in RFA-treated HCC patients.

**Fig 5:**
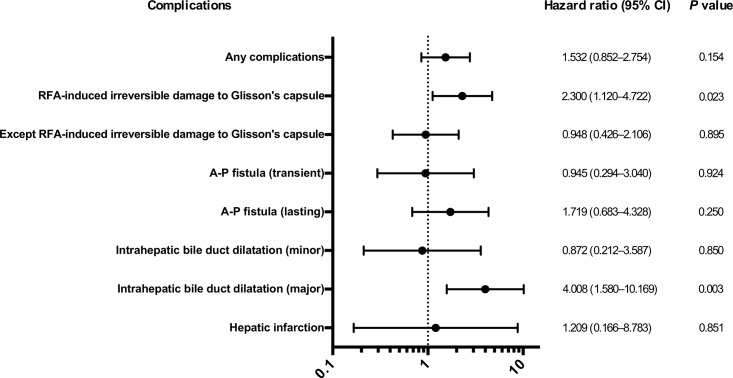
The forest plots according to complications related to RFA in HCC patients.

During the study period, 55 of 170 patients died (median follow-up: 46.7 months). Recurrence-free survival was not significantly different between patients with and without RFA-induced Glisson’s capsule-associated complications (*P* = 0.365). The median OS was significantly worse in patients with RFA-induced Glisson’s capsule-associated complications than in patients lacking Glisson’s capsule-associated complications [52.3 months (95% CI: 34.4–71.7) vs. 95.0 months (95% CI: 73.5–116.5), *P* = 0.021, Fig 4]. The forest plot indicated that RFA-induced Glisson’s capsule-associated complication significantly correlated with a poor prognosis compared with other complications (Fig 5). Multivariate analysis showed that RFA-induced Glisson’s capsule-associated complication was a significant independent factor linked to a poor prognosis as well as being HCV-Ab positive (Table 5). We also analyzed cause of death in patients with and without RFA-induced Glisson’s capsule-associated complications. The rate of the death due to liver dysfunction was significantly higher in patients with RFA-induced Glisson’s capsule-associated complications than in patients without Glisson’s capsule-associated complications (26.6% vs. 6.5%, *P* <0.001).

**Table 5:** Univariate and multivariate analysis of survival in RFA-treated patients with HCC.

Variables	Univariate analysis		*P*	Multivariate analysis		*P*
	Hazard ratio	95% CI		Hazard ratio	95% CI	
**Gender, male**	1.188	0.670–2.108	0.556			
**Age, >74 years**	1.547	0.910–2.633	0.107			
**HBs-Ag positive**	0.378	0.092–1.555	0.178			
**HCV-Ab positive**	2,878	1.232–6.724	0.015	2.840	1.216–6.636	0.016
**Alcohol abuse**	0.751	0.271–2.082	0.581			
**Child—Pugh B**	1.209	0.545–2.683	0.640			
**Tumor size and number, >20mm, Multiple**	1.300	0.745–2.267	0.356			
**AFP, > 100 ng/mL**	1.021	0.513–2.034	0.952			
**RFA-induced Glisson’s capsule-associated complication**	2.300	1.120–4.722	0.023	2.247	1.094–4.613	0.027
**Complications except RFA-induced Glisson’s capsule-associated complication**	0.948	0.426–2.106	0.895			

Abbreviations: HBs-Ag: Hepatitis B surface antigen, HCV-Ab: Hepatitis C virus antibody, AFP: α-fetoprotein.

## Discussion

In this study, we aimed to assess the effect of RFA-induced Glisson’s capsule-associated complication on HCC prognosis. Our results show that HCC patients with RFA-induced Glisson’s capsule-associated complications are at a higher risk for liver failure and a poor prognosis compared with patients without Glisson’s capsule-associated complications.

It is well known that lasting AP fistula develops portal hypertension and causes several typical hepatic symptoms such as ascites and varices [16, 17]. Fig 5 indicates that lasting AP fistula was strongly correlated with a poor prognosis compared with transient AP fistula. Kondo et al. reported that major intrahepatic bile-duct dilatation due to RFA was the cause of decreased albumin and increased bilirubin and poor prognosis in HCC patients [10]. This study also noted that minor intrahepatic bile-duct dilatation did not affect HCC prognosis, a finding corroborated in our study (Fig 5). Hepatic infarction has been shown to occur when hepatic arterial and portal vein flow are both compromised, leading to segmental liver necrosis and failure [18].

In our study, almost half of our patients with AP fistulae after RFA possessed unrecoverable fistulae. AP fistula occurs secondary to percutaneous liver procedures, such as liver biopsy, transhepatic bile-duct drainage, and RFA [19–22]. However, to the best of our knowledge, AP fistula wasn’t included as a complication due to RFA in previous studies [6, 23–24]. This might be because they highlighted complications immediately after RFA. Our study reviewed not only radiological assessments immediately after RFA but also follow-up examinations. Thus, we could rigorously evaluate the landscape of AP fistulae in relation to RFA. Our study also noted that AP fistulae occur not only adjacent to, but also distantly from the tumor, and importantly, AP fistulae seem to develop due to either or both sticking electrodes directly and terminal energy at the time of ablation. A previous report indicated that the rates of minor and major bile-duct dilatation were 11.9% and 3.6%, respectively [10]. These rates were in agreement with our results. Additionally, other RFA complications such as liver abscess, and dissemination were similar to previous studies [23–25].

In this study, we selected RFA-induced Glisson’s capsule-associated complications as noted above, although they did not necessarily cover all possible complications. Portal vein thrombosis, intra-abdominal hemorrhage, hemobilia, and arteriovenous (AV) fistula were excluded from this analysis for the following reasons. First, one previous study reported that RFA-related portal vein thrombosis is rarely progressive with little influence on liver function [26]. Second, intra-abdominal hemorrhage, hemobilia and AV fistula were transient and asymptomatic and required no additional treatment such as blood transfusion in the patients analyzed in this study.

As progressive disease affects liver function in HCC patients, it is difficult to tease out and dissect the cause of liver failure. We assessed the cumulative incidence of liver failure before HCC stage progression and found that early-stage patients with RFA-induced Glisson’s capsule-associated complications had significant deterioration of liver function. Thus, this method of analysis clearly showed the impact of Glisson’s capsule-associated complication on liver function.

Shifting the focus on the effect of RFA-induced Glisson’s capsule-associated complication on HCC prognosis and survival has shed new light on technical gaps in the execution of RFA. Herein, we have demonstrated the dire consequences of such a Glisson’s capsule-associated complication. In addition, our study highlights the importance of enhancing efforts to avoid RFA-induced Glisson’s capsule-associated complication when conducting RFA. Prophylactic cooling of the bile-duct during RFA via intraductal perfusion of saline or 5% dextrose and use of an endoscopic nasobiliary drainage (ENBD) tube has been shown to decrease the incidence of biliary injuries [27–31] Although ENBD tube usage carries risks of complications, such as bleeding, gastrointestinal perforation, and pancreatitis, this may be a viable option to decrease the chances of RFA-induced Glisson’s capsule-associated complications. However, development of techniques that will drastically decrease RFA-induced Glisson’s capsule-associated complications without introducing other kinds of complications is expected.

In conclusion, early-stage HCC patients with RFA-induced Glisson’s capsule-associated complications may have higher risks in poor prognosis. Until there are more effective alternative therapies, clinicians performing RFA should ensure proper technique when cauterizing tumors to mitigate the risk of Glisson’s capsule-associated complications.

## Supporting Information

S1 Table The rate of RFA-induced Glisson’s capsule-associated complication in each subsegment. (PDF)

S2 Table Univariate and multivariate analysis of risk factor for RFA-induced Glisson’s capsule-associated complication per tumor. (PDF)
