# Supplementary material for: Impact of Radiofrequency Ablation-Induced Glisson’s Capsule-Associated Complications in Patients with Hepatocellular Carcinoma
Source: PLoS One. 2017 Jan 18;12(1):e0170153. doi: 10.1371/journal.pone.0170153 (PMC5242538; doi:10.1371/journal.pone.0170153)
Supplement: S1 Table — (PDF) [file pone.0170153.s001.pdf]

S1 Table. The rate of RFA-induced Glisson's capsule-associated complication in each subsegment.

Subsegment of the liver	With RFA-induced Glisson's capsule-associated complication (n = 15)	Without RFA-induced Glisson's capsule-associated complication (n = 197)	*P*
**S1** [*n* (%)]	0	0	0.137
**S2** [*n* (%)]	0 (0)	11 (100)	
**S3** [*n* (%)]	3 (15.0)	17 (85)	
**S4** [*n* (%)]	4 (15.4)	22 (84.6)	
**S5** [*n* (%)]	0 (0)	32 (100)	
**S6** [*n* (%)]	1 (2.7)	36 (97.3)	
**S7** [*n* (%)]	2 (6.7)	28 (93.3)	
**S8** [*n* (%)]	5 (8.9)	51 (91.1)	

Abbreviations: RFA, radiofrequency ablation; S, subsegment.
