# Supplementary material for: Impact of Radiofrequency Ablation-Induced Glisson’s Capsule-Associated Complications in Patients with Hepatocellular Carcinoma
Source: PLoS One. 2017 Jan 18;12(1):e0170153. doi: 10.1371/journal.pone.0170153 (PMC5242538; doi:10.1371/journal.pone.0170153)
Supplement: S2 Table — (PDF) [file pone.0170153.s002.pdf]

S2 Table. Univariate and multivariate analysis of risk factor for RFA-induced Glisson's capsule-associated complication per tumor.

Variables	Univariate analysis		*P*	Multivariate analysis		*P*
	Odds ratio	95% CI		Odds ratio	95% CI	
**Gender, male**	1.438	0.441–4.688	0.546			
**Age, >74 years**	0.542	0.179–1.644	0.279			
**HBs-Ag positive**	0.871	0.107–7.087	0.898			
**HCV-Ab positive**	0.880	0.268–2.892	0.833			
**Alcohol abuse**	2.022	0.414–9.864	0.384			
**Child–Pugh B**	0.571	0.72–4.558	0.597			
**Tumor size, >20 mm**	0.886	0.304–2.585	0.825			
**AFP, >100 ng/mL**	1.206	0.323–4.505	0.781			
**S1**	–	–	–			
**S2**	0.000	0.000–	0.999			
**S3**	2.662	0.684–10.364	0.158			
**S4**	2.909	0.853–9.925	0.088			
**S5**	0.000	0.000–	0.999			
**S6**	0.280	0.041–2.524	0.321			
**S7**	0.897	0.192–4.182	0.889			
**S8**	1.441	0.470–4.416	0.552			
**Location, perivascular**	3.636	1.049–12.600	0.042	3.636	1.049-12.600	0.042

Abbreviations: HBs-Ag, hepatitis B surface antigen; HCV-Ab, hepatitis C virus antibody; AFP, α-fetoprotein; S, subsegment.
